# Impact of Coronary Artery Disease and Percutaneous Coronary Intervention on Transcatheter Aortic Valve Implantation

**DOI:** 10.1155/2021/6672400

**Published:** 2021-03-24

**Authors:** Anthony G. Matta, Thibault Lhermusier, Francisco Campelo Parada, Frederic Bouisset, Ronan Canitrot, Vanessa Nader, Stéphanie Blanco, Meyer Elbaz, Jerome Roncalli, Didier Carrié

**Affiliations:** ^1^Department of Cardiology, Rangueil University Hospital, Toulouse, France; ^2^Faculty of Medicine, Holy Spirit University of Kaslik, Jounieh, Lebanon

## Abstract

**Introduction:**

The prevalence of coronary artery disease (CAD) detected in preoperative work-up for transcatheter aortic valve implantation (TAVI) is high. Instead, the management of a concomitant CAD remains unclear. We evaluate the impact of CAD and percutaneous coronary intervention (PCI) on TAVI procedures.

**Materials and Methods:**

A retrospective study was conducted on 1336 consecutive patients who underwent TAVI in Toulouse University Hospital, Rangueil, France. The studied population was divided into 2 groups: CAD-TAVI group and No CAD-TAVI group. Then, the CAD-TAVI group was segregated into 2 subgroups: PCI-TAVI group and No PCI-TAVI group. In-hospital adverse clinical outcomes were assessed in each group.

**Results:**

Pre-TAVI work-up revealed significant CAD in 36% of 1030 patients eligible for inclusion in the study. The overall prevalence of in-hospital death, stroke, major or life-threatening bleeding, minor bleeding, major vascular complications, minor vascular complications, pacemaker implantation, and acute kidney injury was 2.7%, 2.4%, 2.8%, 3.6%, 3.9%, 7.5%, 12.5%, and 2.7%, respectively. Among the studied population, 55% were admitted to the cardiac care unit. No significant statistical difference was observed between groups. *Discussion*. CAD-TAVI population was not more likely to develop in-hospital adverse clinical outcomes post-TAVI procedure compared to others. Also, no significant difference regarding in-hospital death was observed. In parallel, performing PCI prior to TAVI did not increase the risk of in-hospital death and complications. The difference in terms of the distribution of antithrombotic regimen may explain the higher prevalence of bleeding events in the PCI-TAVI group.

**Conclusion:**

This study provides direct clinical relevance useful in daily practice. No negative impact has been attributed to the presence of a concomitant CAD and/or preoperative PCI on the TAVI hospitalization period.

## 1. Introduction

Coronary artery disease (CAD) and degenerative aortic stenosis (AS) are two different cardiovascular entities that frequently coexist and share in common multiple risk factors, pathophysiological mechanisms, and clinical relevance [1, 2]. Transcatheter aortic valve implantation (TAVI) has become a widely used procedure to manage patients with severe AS. In accordance with results from recent trials, TAVI indication was extended from patients at high risk to those at moderate and low risk [3–5]. View the high prevalence of CAD in patients with AS; coronary angiography is routinely performed in pre-TAVI work-up. An incidental diagnosis of significant CAD in patients referred to TAVI is often in our daily practice. Until now, the impact of a concomitant CAD on TAVI procedural outcomes is still a matter of debate and available data in the literature are controversial [6–9]. A few studies found that CAD is a predictor of mortality in the short term while others noticed the absence of significant effects [10]. Indeed, the optimal time for revascularization in patients undergoing TAVI is uncertain and it is the main objective for ongoing trials [11]. In parallel, it remains unclear if the percutaneous coronary intervention (PCI) prior to TAVI may offer additional benefits [11]. Given the paucity of published studies and the lack of strong evidence from randomized clinical trials resulting in the absence of standardized recommendations, the management of CAD in TAVI patients is based on a case-by-case local heart team multidisciplinary decision. Herein, we primarily aim to evaluate the impact of a concomitant CAD revealed by preoperative work-up on post-TAVI in-hospital adverse clinical outcomes. Also, we evaluate the effect of PCI performed prior to TAVI in CAD-TAVI patients.

## 2. Materials and Methods

### 2.1. Study Design and Population

**Figure 1:**
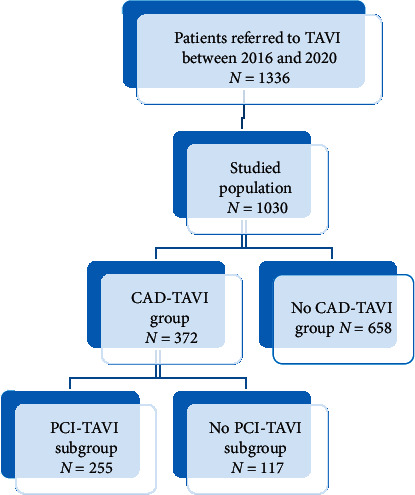
Study flow chart.

An observational retrospective study was conducted on 1336 consecutive patients referred for TAVI at the structural and interventional cardiology department at Toulouse University hospital, Rangueil, France, between January 2016 and March 2020. Patients with a previous history of coronary artery disease [prior myocardial infarction (MI), PCI, or coronary artery bypass graft (CABG)] were excluded from the study as we are interested in CAD revealed by pre-TAVI work-up. The studied population (1030) was divided into 2 groups: CAD-TAVI group including all patients with significant CAD defined by ≥ 50% visual angiographic stenosis in a major coronary vessel versus others (No CAD-TAVI group). Thereafter, the CAD-TAVI group was segregated into 2 subgroups: those treated with PCI within 3 months prior to TAVI (PCI-TAVI subgroup) versus those kept on medical therapy or PCI was postponed to a later date after the TAVI procedure (No PCI-TAVI subgroup) (Figure 1). Strategy for the management of CAD revealed by the preoperative work-up (medical, PCI prior to TAVI or delayed PCI) was based on heart team decision.

### 2.2. Data Collection and End Points

Data concerning baseline characteristics (age, sex), cardiovascular risk factors (diabetes mellitus, systemic hypertension, smoking, dyslipidemia, and BMI), medical treatment (aspirin, P2Y12 inhibitors, and oral anticoagulant), previous medical history (prior MI, PCI, CABG, stroke, and peripheral artery disease), concomitant comorbidities (chronic respiratory disease and atrial fibrillation), pre-TAVI coronary angiogram results (normal, one, two, and three vessels disease), pre-TAVI PCI, and TAVI procedure (indication, approach, and valve types) were collected. In-hospital post-TAVI adverse clinical outcomes were defined as 1-Major Adverse Cerebrovascular and Cardiac Events (MACCE) defined as the composite of death from any cause, 2-Significant complications including bleeding (minor, major, or life-threatening), vascular complications (minor or major), and acute kidney injury defined according to Valve Academic Research Consortium-2 Criteria [12], stroke and pacemaker implantation, and 3-cardiac care unit admission. The primary endpoint is to determine the impact of a concomitant CAD revealed by preoperative work-up on in-hospital post-TAVI adverse clinical outcomes. The secondary endpoint is to evaluate the effect of PCI performed prior to TAVI on postprocedure in-hospital adverse clinical outcomes as defined above.

### 2.3. Statistical Analysis

Categorical variables were summarized as numbers and percentages and continuous variables as means ± standard deviations. Continuous variables were compared with the use of *t*-test, as appropriate, and categorical variables with the use of *χ*^2^ or Fisher's exact test, as appropriate. A stepwise logistic regression analysis adjusted on all variables with *p* value <0.2 in the bivariate analysis comparing the CAD-TAVI group to No CAD-TAVI group was conducted to assess the association between the defined adverse TAVI clinical outcomes and the presence of CAD. Another stepwise logistic regression analysis adjusted on all variables with *p* value < 0.2 in the bivariate analysis comparing PCI-TAVI subgroup to No PCI-TAVI subgroup was conducted to assess the association between the defined adverse TAVI clinical outcomes and PCI prior to TAVI in CAD-TAVI patients. A two-sided *p* value <0.05 was considered of statistical significance. All statistical analyses were carried out by using SPSS version 20.

## 3. Results

Out of 1336 consecutive patients who underwent TAVI, 1030 were eligible for inclusion in the study. 306 were excluded due to the previous history of PCI or CABG. Baseline and demographic characteristics of the studied population are shown in Table 1. The mean age was 84 and 45.2% of patients were males. The baseline left ventricle ejection fraction (LVEF) was 53.7% and the population was at higher surgical risk with a predicted mortality of 6.35 by STS-PROM and of 14.2 by EuroSCORE I. From the studied population, 24% did not receive any antithrombotic treatment, 23.8% received single-antiplatelet therapy (aspirin or clopidogrel), 16.6% received dual-antiplatelet therapy (aspirin + clopidogrel or ticagrelor), 27% were on an anticoagulant therapy alone (antivitamin K “AVK” or direct oral anticoagulant “DOAC”), and 8.6% were in combination with antiplatelet therapy (aspirin or clopidogrel). Most TAVI procedures were performed through transfemoral access (93.9%) and balloon-expandable valves (Edwards Sapien) were implanted in 54.4% and self-expanding valves (Corevalve Evolut Pro, Corevalve Evolut *R*, ACURATE) in 45.6% of patients. Overall, the prevalence of death, stroke, major or life-threatening bleeding, minor bleeding, major vascular complications, minor vascular complications, pacemaker implantation, and acute kidney injury were 2.7%, 2.4%, 2.8%, 3.6%, 3.9%, 7.5%, 12.5%, and 2.7%, respectively. Almost 55% of the population were admitted to the cardiac care unit. It is worthy to mention that myocardial infarction was not observed during hospitalization in all groups. The prevalence of significant CAD revealed incidentally in TAVI preoperative work-up was 36% (372/1030). Then, the population was divided into 2 groups: CAD-TAVI group (*N* = 372) and No CAD-TAVI group (*N* = 658) based on the presence or absence of CAD in pre-TAVI coronary angiograms.

The bivariate analysis has shown significant difference at 0.2 level between the two groups in terms of the distribution of male gender, diabetes mellitus, dyslipidemia, previous history of peripheral artery disease, antithrombotic regimen (dual-antiplatelet and oral anticoagulant combined to antiplatelet), and prosthesis types (balloon-expandable valves) which were more common in CAD-TAVI group while a significant difference was also noted in the repartition of chronic respiratory disease, atrial fibrillation, and antithrombotic therapy (nothing, single antiplatelet, and anticoagulation alone) which were more frequent in No CAD-TAVI group. Except for major or life-threatening bleeding (3.8% vs. 2.3%, *p*=0.16), there are no significant difference between the CAD-TAVI group compared to No CAD-TAVI group regarding death (3% vs. 2.6%, *p*=0.72), stroke (2.4% vs. 2.4% *p*=0.99), major (4.6% vs. 3.5%, *p*=0.16) and minor vascular complications (6.7% vs. 7.9%, *p*=0.48), minor bleeding (3.8% vs. 3.5%, *p*=0.82), pacemaker implantation (13.7% vs. 11.9%, *p*=0.38), acute kidney injury (2.2% vs. 3%, *p*=0.39), and CCU admission (57% vs. 54%, *p*=0.34) (Table 2). After adjusting for confounding variables listed above, the multivariate logistic regression showed that the population of the CAD-TAVI group was not more likely to develop in-hospital post-TAVI adverse clinical outcomes including death (OR = 2.28 95%CI [0.73; 7.1]), major or life-threatening bleeding (OR = 0.56 95%CI[1.6; 2]), major vascular complications (OR = 1.09 95%CI[0.36; 3.27]), stroke (OR = 0.56 95%CI = [0.14; 2.26]), pacemaker implantation (OR = 1.05 95%CI = [0.55; 1.99]), minor vascular complications (OR = 1.09 95%CI = [0.36; 3.27]), minor bleeding (OR = 0.94 95%CI = [0.3; 2.9]), acute kidney injury (OR = 0.93 95%CI = [0.29; 3]), and CCU admission (OR = 1.38 95%CI = [0.91; 2.1]).

The angiographic characteristics of the CAD-TAVI group are presented in Table 3. The rate of three-vessel, two-vessel, and one vessel disease were 9.7%, 25.5%, and 64.8%, respectively. The left anterior descending artery was the most common affected coronary artery (66%) followed by the right coronary artery (44%), left circumflex (26%), and left main (9.7%). Overall, PCI was performed in 68.5% of the CAD-TAVI group. Baseline and demographic characteristics for both subgroups (PCI-TAVI group vs. No PCI-TAVI group) are presented in Table 4. The bivariate analysis has shown a significant difference at 0.2 level between the two subgroups in terms of distribution of smoking, atrial fibrillation, chronic respiratory disease, and previous history of peripheral artery disease which were more frequent in the NO PCI-TAVI subgroup. Also, antithrombotic regimens [dual-antiplatelet (aspirin + clopidogrel or ticagrelor) and oral anticoagulant (AVK or DOAC) combined to antiplatelet (aspirin or clopidogrel)] were significantly more common in the PCI-TAVI subgroup. Except for major or life-threatening bleeding (4.7% vs. 1.7%, *p*=0.14), there are no significant difference between the PCI-TAVI subgroup compared to the No PCI-TAVI subgroup regarding death (2.8% vs. 3.4%, *p*=0.75), stroke (2.4% vs. 2.5% *p*=0.93), major (5.1% vs. 3.4%, *p*=0.44) and minor vascular complications (5.9% vs. 8.4%, *p*=0.37), minor bleeding (4% vs. 3.4%, *p*=0.78), pacemaker implantation (13% vs. 15.1%, *p*=0.58), acute kidney injury (1.6% vs 3.4%, *p*=0.27), and CCU admission (53% vs. 65.5%, *p*=0.22) (Table 5). After adjusting for confounding variables, the multivariate logistic regression showed that the population of the PCI-TAVI group was not more likely to develop in-hospital post-TAVI adverse clinical outcomes including death (OR = 1.3 95%CI [0.2; 8.4]), major or life-threatening bleeding (OR = 4.7 95%CI[0.37; 60]), major vascular complications (OR = 0.61 95%CI[0.08; 4.38]), stroke (OR = 0.8 95%CI = [0.11; 5.82]), pacemaker implantation (OR = 0.41 95%CI = [0.14; 1.22]), minor vascular complications (OR = 0.79 95%CI = [0.24; 2.62]), minor bleeding (OR = 3.15 95%CI = [0.7; 14.17]), acute kidney injury (OR = 0.45 95%CI = [0.04; 5]), and CCU admission (OR = 0.65 95%CI = [0.32; 1.35]).

## 4. Discussion

Diagnosing concomitant significant CAD in patients with severe AS who underwent TAVI is an important daily concern. This fact was confirmed by this study that showed a high prevalence of CAD (36%) revealed by the pre-TAVI coronary angiography. However, the impact of the presence of CAD on TAVI procedures remains unclear and controversial [6–9]. Also, the management of CAD in the TAVI population including the appropriate indication and timing for PCI is not well defined. While CABG is definitely recommended for its attributable survival benefit in CAD patients referred for aortic valve replacement [13, 14], the 2017 European guidelines recommended that PCI should be considered in CAD-TAVI patients views the low level of evidence from clinical trials in regard to beneficial outcomes of PCI [15].

The results of this study showed that the CAD population selected via preintervention coronary angiography was not predisposed to develop postprocedural in-hospital adverse clinical outcomes compared to those with patent coronary arteries. A similar result was found by the UK TAVI registry [16] and the advance study [17]. Also, an analysis from the German registry showed that coexisting CAD had no impact on overall survival in TAVI patients after adjusting for confounders [18]. In opposition to previously published studies conducted on patients with prior history of coronary artery disease defined by a previous PCI or CABG, this study was interested exclusively in CAD detected by preoperative work-up. In parallel, no effect for PCI performed prior to TAVI in CAD-TAVI population on death, vascular complications, bleeding, acute kidney injury, pacemaker implantation, and CCU admission was revealed by this study. Indeed, a higher prevalence of major bleeding events probably explained by the difference in antithrombotic regimen was noted in the CAD-TAVI group and PCI-TAVI subgroup but it became no longer significant after adjusting for confounders. Physicians prefer to do PCI prior to TAVI for 2 main reasons. First, clinical manifestations of CAD and AS may overlap and inducible ischemia tests that may differentiate the culprit role of CAD from AS are contra-indicated in patients with severe AS. Then, PCI prior to TAVI may resolve the symptoms requiring a reevaluation for TAVI indication. Secondly, TAVI intervention may modify the access to the coronary arteries making it technically more difficult to do PCI. Lastly, our study emphasized that performing PCI in the CAD-TAVI population was not associated with in-hospital adverse clinical outcomes. Instead, it is up to the physician to assess the proper indication for PCI knowing that it does not lead to any relevant cardiovascular improvement when performed in TAVI patients [11] or/and patients with stable CAD [19, 20].

To conclude, we believe that our study provides direct clinical relevance useful in daily practice. The absence of negative impact attributed to a concomitant CAD on TAVI procedure and of positive effect attributed to PCI on post-TAVI cardiovascular outcomes warrant a reevaluation for the utility of preoperative coronary angiography in such patients with stable angina and no previous history of CAD (previous MI, PCI, or CABG) in the era of coronary computed tomography angiography. Chieffo et al. showed that coronary angiography was only necessary on top of computed tomography angiography in 22% from overall studied pre-TAVI work-up [21]. Knowing that PCI did not add benefits to cardiovascular outcomes in patients with stable CAD, it must be preserved only for patients with unstable angina, serious comorbidities, and those at high risk for perprocedural complications and subsequent hemodynamic instability.

### 4.1. Study Limitations

The retrospective nonrandomized study design may predispose to selection bias. PCI versus no PCI was chosen based on the Heart Team's clinical judgment in the best interest of the patient at the time of the procedure. CAD plus no PCI group can include 70% and 50% lesion while CAD plus PCI group can only include 70% or FFR positive 50% lesion. The study is limited on periprocedural and in-hospital outcomes and no data were provided on long-term follow-up.

## 5. Conclusion

To summarize, CAD revealed in pre-TAVI work-up was not associated with significant in-hospital adverse clinical outcomes. Also, performing PCI when needed did not increase the risk of death or procedural complications. A future head-to-head prospective comparative study investigating the impact of CAD and PCI on short- and long-term TAVI procedures is required leading to establishing standardized guidelines selecting in whom pre-TAVI coronary angiography and PCI are of potential benefits.

## Figures and Tables

**Table 1:** Baseline characteristics.

	Whole population	CAD group	No CAD group	*p* value CAD versus no CAD
	*N* = 1030	*N* = 372	*N* = 658	
Age	84.3 ± 7	84.2 ± 7	84.6 ± 7	0.35
Male	466 (45.2)	196 (52.7)	270 (41)	<0.001
Hypertension	708 (68.7)	253 (68)	455 (69.1)	0.7
Diabetes mellitus	264 (74.4)	108 (29)	156 (23.7)	0.05
Dyslipidemia	410 (39.8)	175 (47)	235 (35.7)	<0.001
Smoking	26 (2.5)	6 (1.6)	20 (3)	0.16
BMI	26.2 ± 5	26.2 ± 4.9	30.7 ± 4.7	0.27
Chronic respiratory disease	193 (18.7)	61 (16.4)	132 (20.1)	0.14
Atrial fibrillation	394 (38.3)	130 (34.9)	264 (40.1)	0.1
Prior stroke	110 (10.7)	41 (11)	69 (10.5)	0.78
Prior PAD	69 (6.7)	31 (8.3)	38 (5.8)	0.11
EuroScore1	14.2 ± 9.9	14.3 ± 10	14 ± 10	0.84
STS-PROM	6.35 ± 4.7	6.3 ± 4.7	5.7 ± 5.5	0.68
Baseline LVEF	53.7 ± 11.8	53.2 ± 12.3	53.7 ± 11.5	0.07

*Antithrombotic treatment*				
None	247 (24)	35 (9.4)	212 (32.2)	<0.001
Single-antiplatelet therapy	245 (23.8)	65 (17.5)	180 (27.4)	
Dual-antiplatelet therapy	171 (16.6)	156 (41.9)	15 (2.3)	
Anticoagulation therapy	278 (27)	49 (13.2)	229 (34.8)	
Antiplatelet + anticoagulation	89 (8.6)	67 (18)	22 (3.3)	

*TAVI approach*				
Transfemoral	967 (93.9)	349 (93.8)	618 (93.9)	0.22
Transaortic	54 (5.2)	18 (4.8)	36 (5.5)	
Transapical	2 (0.2)	0 (0)	2 (0.3)	
Transsubclavian	5 (0.5)	4 (1.1)	1 (0.2)	
Transcarotid	2 (0.2)	1 (0.3)	1 (0.2)	

Valve in valve	35 (3.4)	14 (3.8)	21 (3.2)	0.62

Prosthesis type				
Ballon expandable	560 (54.4)	217 (58.3)	343 (52.1)	0.05
Self-expanding	470 (45.6)	155 (41.7)	315 (47.9)	

Post-TAVI LVEF	54.9 ± 10.7	54.7 ± 10.7	55.6 ± 10.9	0.85

^*∗*^CAD = coronary artery disease. BMI = body mass index. PAD = peripheral artery disease. LVEF = left ventricle ejection fraction. EuroScore = European System for Cardiac Operative Risk Evaluation. STS-PROM = Society of Thoracic Surgeons Predicted Risk of Mortality. TAVI = transcatheter aortic valve implantation. CCU = cardiac care unit. ^*∗*^TAVI: transcatheter aortic valve implantation. CAD: coronary artery disease. PCI: percutaneous coronary intervention.

**Table 2:** Procedural adverse clinical outcomes stratified by the presence of concomitant coronary artery disease (CAD).

	Whole population	CAD group	No CAD group	*p* value CAD versus no CAD
	*N* = 1030	*N* = 372	*N* = 658	
Death, N(%)	28 (2.7)	11 (3)	17 (2.6)	0.72

Significant complications, N(%)				
Major or life-threatening bleeding	29 (2.8)	14 (3.8)	15 (2.3)	0.16
Minor bleeding	37 (3.6)	14 (3.8)	23 (3.5)	0.82
Major vascular complication	40 (3.9)	17 (4.6)	23 (3.5)	0.39
Minor vascular complication	77 (7.5)	25 (6.7)	52 (7.9)	0.48
Stroke	25 (2.4)	9 (2.4)	16 (2.4)	0.99
Acute kidney injury	28 (2.7)	8 (2.2)	20 (3)	0.39
Pacemaker implantation	129 (12.5)	51 (13.7)	78 (11.9)	0.38

CCU admission, N(%)	567 (55)	212 (57)	355 (54)	0.34

**Table 3:** Angiographic characteristics of CAD-TAVI group (*N* = 372).

	N (%)
*Vessel disease*	
1	241 (64.8)
2	95 (25.5)
3	36 (9.7)

*Lesion distribution*	
LM	36 (9.7)
LAD	246 (66.1)
LCX	97 (26.1)
RCA	164 (44.1)

PCI	255 (68.9)

^*∗*^LM = left main. LAD = left anterior descending. LCX = left circumflex. RCA = right coronary artery. PCI = percutaneous coronary intervention.

**Table 4:** Baseline characteristics of PCI subgroup in CAD-TAVI population.

	CAD group	PCI group	No PCI group	*p* value PCI versus no PCI
	*N* = 372	*N* = 255	*N* = 117	
Age	84 ± 7	84 ± 6.7	84 ± 7.5	0.21
Male	196 (52.7)	132 (52.2)	64 (53.8)	0.77
Hypertension	253 (68)	172 (68)	81 (68.1)	0.98
Diabetes mellitus	108 (29)	77 (30.4)	31 (26.1)	0.38
Dyslipidemia	175 (47)	124 (49)	51 (42.9)	0.26
Smoking	6 (1.6)	1 (0.4)	5 (4.2)	0.007
BMI	26.2 ± 4.9	26.2 ± 4.8	26.1 ± 5.1	0.57
Chronic respiratory disease	61 (16.4)	36 (14.2)	25 (21)	0.1
Atrial fibrillation	130 (34.9)	81 (32)	49 (41.2)	0.08
Prior stroke	41 (11)	29 (11.5)	12 (10.1)	0.69
Prior PAD	31 (8.3)	17 (6.7)	14 (11.8)	0.1
EuroScore1	14.3 ± 10	14.1 ± 10.2	14.6 ± 9.2	0.57
STS-PROM	6.3 ± 4.7	6.6 ± 4.9	5.8 ± 4.3	0.24
Baseline LVEF	53.2 ± 12.3	53.9 ± 11.6	53.4 ± 12.1	0.8

*Antithrombotic treatment*				
None	35 (9.4)	11 (4.3)	24 (20.2)	<0.001
Single-antiplatelet therapy	65 (17.5)	23 (9.1)	42 (35.3)	
Dual-antiplatelet therapy	156 (41.9)	151 (59.7)	5 (4.2)	
Anticoagulation therapy	49 (13.2)	6 (2.4)	43 (36.1)	
Antiplatelet + anticoagulation	67 (18)	62 (24.5)	5 (4.2)	

TAVI approach				
Transfemoral	349 (93.8)	237 (93.7))	112 (94.1)	0.9
Transaortic	18 (4.8)	12 (4.7)	6 (5)	
Transsubclavian	0 (0)	3 (1.2)	1 (0.8)	
Transcarotid	4 (1.1)	1 (0.4)	0 (0)	
	1 (0.3)			

Valve in valve	14 (3.8)	9 (3.6)	5 (4.2)	0.76

*Prosthesis type*				
Ballon expandable	217 (58.3)	151 (59.7)	66 (55.5)	0.44
Self-expanding	155 (41.7)	102 (40.3)	53 (44.5)	

Post-TAVI LVEF	54.7 ± 10.7	54.9 ± 10.6	54.8 ± 10.9	0.9

^*∗*^CAD = coronary artery disease. PCI = percutaneous coronary intervention. BMI = body mass index. PAD = peripheral artery disease. EuroScore = European System for Cardiac Operative Risk Evaluation. STS-PROM=Society of Thoracic Surgeons Predicted Risk of Mortality. TAVI = transcatheter aortic valve implantation. CCU = cardiac care unit.

**Table 5:** Procedural adverse clinical outcomes in CAD-TAVI group stratified by the performance of PCI.

	CAD group	PCI group	No PCI group	*p* value PCI versus no PCI
	*N* = 372	*N* = 255	*N* = 117	
Death, N (%)	11 (3)	7 (2.8)	4 (3.4)	0.75

*Significant complications, N(%)*				
Major or life-threatening bleeding	14 (3.8)	12 (4.7)	2 (1.7)	0.14
Minor bleeding	14 (3.8)	10 (4)	4 (3.4)	0.78
Major vascular complication	17 (4.6)	13 (5.1)	4 (3.4)	0.44
Minor vascular complication	25 (6.7)	15 (5.9)	10 (8.4)	0.37
Stroke	9 (2.4)	6 (2.4)	3 (2.5)	0.93
Acute kidney injury	8 (2.2)	4 (1.6)	4 (3.4)	0.27
Pacemaker implantation	51 (13.7)	33 (13)	18 (15.1)	0.58

CCU admission, N (%)	212 (57)	134 (53)	78 (65.5)	0.22

^*∗*^CAD = coronary artery disease. PCI = percutaneous coronary intervention. CCU = cardiac care unit.

## Data Availability

The data are available upon request to the authors.
